# A Predictive Bioengineering Model of Dental Implant Instability in Systemic Bone Disorders: A Periotest-Based Analysis

**DOI:** 10.3390/bioengineering13030297

**Published:** 2026-03-03

**Authors:** Liliana Sachelarie, Ramona Feier, Corina-Laura Ștefănescu, Mircea Grigorian, Rodica-Maria Murineanu, Zaharia Agripina, Loredana Liliana Hurjui

**Affiliations:** 1 Department of Dental Medicine, Apollonia University, 700511 Iasi, Romania; lisachero@yahoo.com; 2 Department of Dental Medicine, Dimitrie Cantemir University, 540545 Târgu Mureș, Romania; dr.ramonafeier@yahoo.ro; 3 Faculty of Medicine and Pharmacy, University Ovidius, Constanta, Mamaia Boulevard, 900527 Constanta, Romania; rodicamurineanu@365.univ-ovidius.ro; 4 Private Dental Practice, 900162 Constanta, Romania; agrizaharia@yahoo.com; 5 Faculty of Medicine, Grigore T. Popa University of Medicine and Pharmacy Iasi, University Street 16, 700115 Iasi, Romania; loredana.hurjui@umfiasi.ro

**Keywords:** dental implant instability, implant micromotion, biomechanical parameters, interface stiffness

## Abstract

(1) Background: Dental implant instability represents a dynamic biomechanical process influenced by functional loading, peri-implant bone stiffness, and systemic conditions affecting bone metabolism. In patients with systemic bone disorders, altered material properties and impaired remodeling may reduce effective implant–bone interface stiffness, potentially increasing micromotion beyond what is detectable by conventional clinical indicators. The aim of this study was to develop and evaluate a predictive bioengineering model of implant instability based on Periotest-derived dynamic measurements. (2) Methods: A retrospective analysis was performed on 79 dental implants placed in patients with and without systemic bone disorders. Implant micromotion was quantified using Periotest values (PTVs). Linear and logistic regression analyses were applied to model the relationship between systemic bone status, implant location, and biomechanical instability (defined as PTV > +2.0). A load–stiffness–micromotion framework was used to provide mechanical interpretation of the findings. (3) Results: Implants placed in patients with systemic bone disorders exhibited significantly higher Periotest values compared to controls (+2.1 ± 1.3 vs. −0.4 ± 1.1; mean difference 2.5 PTV units, 95% CI 1.97–3.04; *p* < 0.001). High-risk biomechanical instability (PTV > +2.0) was observed in 46% of implants in the systemic group compared to 9% in controls. Multivariable logistic regression demonstrated that systemic bone disorders were independently associated with a 2.6-fold increase in the odds of high-risk instability after adjustment for implant location. The observed instability pattern was consistent with reduced effective peri-implant stiffness in systemically compromised bone. (4) Conclusions: Dental implant instability in systemically compromised patients can be interpreted as a load–stiffness imbalance at the implant–bone interface. The proposed predictive bioengineering framework links dynamic Periotest measurements with mechanical modeling and systemic bone status, enabling quantitative risk stratification beyond static stability assessments.

## 1. Introduction

Despite the high success rates reported for dental implant therapy, implant instability remains a clinically relevant complication, particularly in patients affected by systemic bone disorders. Clinical success in implant dentistry cannot be defined solely by implant survival, as biomechanical deterioration at the implant–bone interface may precede radiographic bone loss or overt clinical failure. Implant stability is therefore a dynamic process that reflects the balance between mechanical anchorage, peri-implant bone quality, and biological remodeling over time [1,2]. While early implant failures are often attributed to surgical inaccuracies or prosthetic overload, delayed or progressive instability is more frequently associated with compromised bone metabolism and impaired host bone adaptive capacity [3].

Systemic bone impairment, such as osteoporosis and other metabolic or inflammatory conditions, is characterized by reduced bone mass, altered microarchitecture, and dysregulated remodeling processes [4,5]. In the context of implant dentistry, systemic bone conditions reported to influence implant stability and survival include osteoporosis, osteopenia, osteomalacia, Paget’s disease, hyperparathyroidism, and corticosteroid-induced bone loss, all of which may alter bone remodeling dynamics and mechanical competence at the implant–bone interface. These systemic alterations do not merely affect bone quantity, but also influence bone material properties and mechanical behavior. As a consequence, peri-implant bone in systemically compromised patients may exhibit reduced stiffness, increased energy dissipation, and diminished load-bearing capacity, resulting in a higher susceptibility to implant micromotion under functional loading [6]. Despite increasing clinical awareness of these associations, the underlying biomechanical mechanisms linking systemic bone impairment to implant instability remain insufficiently explored.

Most existing studies in implant dentistry focus on implant stability as a static or early outcome, commonly assessed using insertion torque measurements or resonance frequency analysis (RFA) [7,8]. These parameters are primarily used to evaluate primary stability at the time of implant placement and to guide immediate or early loading protocols. While such methods provide valuable information regarding initial mechanical anchorage, they offer limited insight into the progressive biomechanical degradation of the implant–bone interface over time, particularly in patients with systemic compromise. Consequently, implant instability is often detected only at advanced stages, when corrective interventions are limited and implant prognosis is already compromised.

In contrast, implant instability should be regarded as a time-dependent biomechanical process driven by the interaction between functional loading, peri-implant bone stiffness, and biological adaptation [9]. Implant micromotion at the bone–implant interface plays a central role in this process, as excessive micromotion may interfere with normal osseointegration, promote fibrous tissue formation, and disrupt long-term biomechanical equilibrium [10]. Importantly, micromotion-related alterations may remain subclinical for extended periods, underscoring the need for assessment methods that capture early biomechanical changes before irreversible damage occurs.

Periotest assessment provides an established, non-invasive method for quantifying the dynamic response of the implant–bone interface, reflecting implant micromotion and energy dissipation rather than static stability alone [11,12]. Unlike conventional stability measures that primarily characterize initial fixation, Periotest-derived values offer insight into interface damping behavior under impact loading conditions. Although Periotest values have been previously reported in various clinical contexts, their integration into mechanistically informed predictive frameworks remains limited. Most studies employ Periotest measurements descriptively, without explicitly linking them to underlying biomechanical mechanisms or systemic bone-related modifiers.

The present study adopts a bioengineering-oriented perspective, proposing a predictive framework in which dental implant instability is interpreted as the biomechanical outcome of the interaction between functional loading, peri-implant stiffness, and biological adaptation processes. From a mechanical standpoint, implant micromotion reflects the balance between applied forces and interface resistance, which may be altered in the presence of systemic bone disorders.

The aim of this study was to investigate implant instability by analyzing Periotest-derived micromotion values within this load–stiffness–adaptation framework in patients with and without systemic bone disorders. Rather than introducing a new diagnostic modality, the study provides an integrative mechanistic interpretation that links established clinical stability measurements with systemic bone status and anatomical factors in a structured predictive model. This approach aims to support earlier identification of instability risk and enhance clinical decision-making in patients with systemic compromise.

## 2. Materials and Methods

### 2.1. Study Design and Ethical Considerations

This study was conducted as a retrospective observational clinical investigation based on data obtained from routine dental care at a single clinical center. Data were collected from implants placed and evaluated between January 2019 and December 2023. Consecutive eligible cases meeting the predefined inclusion and exclusion criteria were included in the analysis. No additional diagnostic or therapeutic procedures were introduced for research purposes, and patient management was not altered in any way. All Periotest measurements were performed as part of standard clinical evaluation and subsequently analyzed for research purposes.

The study followed the principles of the Declaration of Helsinki. Ethical review and approval were not required due to the retrospective observational design and the use of fully anonymized data obtained from routine clinical practice, in accordance with applicable national regulations governing non-interventional research.

### 2.2. Study Population

The study included 79 dental implants placed in adult patients who underwent implant-supported oral rehabilitation as part of routine clinical care. Patients were recruited from routine clinical practice and classified into two groups based on the presence or absence of systemic bone disorders.

The systemic bone disorder group primarily consisted of patients with physician-diagnosed osteoporosis. Other documented metabolic bone conditions affecting bone turnover were recorded when present; however, due to sample size constraints, systemic bone disorders were analyzed as a composite category. When available, prior densitometry reports or information regarding osteoporosis-related treatment were noted; however, independent verification of bone mineral density values was not performed within the present retrospective design. In cases where other metabolic bone conditions were documented, these were included within the systemic category. Due to sample size considerations and clinical heterogeneity, systemic bone disorders were analyzed as a composite group without stratification by severity.

Eligible participants were adults aged 18 years or older who presented at least one dental implant suitable for stability assessment. Only implants placed in healed sites and allowing standard clinical evaluation were included in the analysis. Patients were excluded if they presented with an acute infection at the implant site at the time of evaluation, had a history of radiotherapy in the head and neck region, or had uncontrolled systemic conditions other than bone disorders. In addition, implants showing clinical mobility at the time of examination were excluded to ensure that the analysis focused on subclinical biomechanical behavior rather than overt implant failure.

### 2.3. Implant Characteristics

All implants included in the study were commercially available dental implants placed according to standard surgical protocols. Implant placement sites (maxilla or mandible) and clinical status at the time of evaluation were recorded. No experimental implant designs, surface treatments, or surgical techniques were used, ensuring that all observations reflected routine clinical conditions.

### 2.4. Implant Stability Assessment

Implant stability was assessed using the Periotest, a non-invasive method that quantifies the dynamic mechanical response of the implant–bone interface. Periotest measurements were performed as part of routine clinical assessment using a standardized Periotest device, following the manufacturer’s instructions.

During measurement, the handpiece was positioned perpendicular to the implant-supported restoration or healing abutment, maintaining consistent contact conditions and angulation. For each implant, three consecutive measurements were obtained under standardized clinical conditions, and the mean value was used for subsequent statistical analysis to minimize measurement variability. All assessments were performed by the same experienced clinician to reduce inter-operator variability. The device was calibrated according to the manufacturer’s recommendations prior to use.

The primary outcome variable was the Periotest Value (PTV), expressed as a numerical score reflecting interface damping and implant micromotion. Higher PTVs indicate greater mechanical mobility and reduced interface stiffness, whereas lower PTVs indicate improved biomechanical stability.

### 2.5. Biomechanical Interpretation Framework

In the present study, Periotest values were not interpreted as isolated clinical indicators but rather as proxy measures of implant micromotion and dynamic interface behavior. From a biomechanical perspective, implant micromotion is influenced by the interaction between functional loading, peri-implant bone stiffness, and biological adaptation processes.

Systemic bone disorders were considered modifiers of peri-implant bone mechanical properties, potentially reducing effective stiffness and increasing energy dissipation at the implant–bone interface. This conceptual framework allowed implant instability to be analyzed as a time-dependent biomechanical phenomenon, reflecting the combined influence of mechanical and biological factors rather than a binary stable–unstable outcome. Within this framework, implant instability was assessed using Periotest-derived micromotion values (PTVs) as the primary biomechanical indicator, together with systemic bone disorder status, implant location, and their interaction in the predictive modeling approach. The +2.0 threshold was selected based on previously reported clinical interpretations of Periotest value ranges, in which values exceeding +2 are generally associated with reduced implant stability and increased risk of micromotion. While not universally standardized, this cutoff is commonly used in clinical practice to differentiate stable from potentially unstable implants and was therefore adopted for risk stratification in the present predictive framework. From a biomechanical perspective, the Periotest measurement reflects the dynamic impact response of the implant–bone system, which can be approximated as a mass–spring–damper structure. In systemically compromised bone, reduced effective peri-implant stiffness (k) and altered damping properties increase micromotion amplitude under identical loading impulses, resulting in higher recorded PTVs.

### 2.6. Statistical Analysis

Statistical analysis was performed at the implant level. Descriptive statistics were used to summarize Periotest values for all implants included in the study. Continuous variables were expressed as mean ± standard deviation (SD), while categorical variables were reported as absolute numbers and percentages. Comparative analyses were conducted to evaluate differences in implant stability between patients with and without systemic bone disorders. Group comparisons of Periotest values were performed using an independent-samples t-test, or the Mann–Whitney U test when normality assumptions were not met.

Periotest values (PTVs) were further analyzed using a linear regression framework, including systemic bone disorder status (B), implant location (L), and their interaction term (B·L), with PTV as the dependent variable. This analysis allowed evaluation of the independent and combined effects of systemic and local factors on implant micromotion.

For predictive modeling, high-risk biomechanical instability was defined as a Periotest value greater than +2.0 (PTV > +2.0; Y = 1), whereas implants with PTV ≤ +2.0 were classified as non-high-risk (Y = 0). A multivariable logistic regression model was applied to estimate the probability of high-risk instability as a function of systemic bone disorder status (B: 1 = present; 0 = absent) and implant location (L: 1 = maxilla; 0 = mandible). Model coefficients (α) were estimated using maximum likelihood, and results were reported as odds ratios (OR = e^α) with corresponding 95% confidence intervals (CIs). Statistical significance was set at *p* < 0.05. All analyses were performed using standard statistical software.

The validity of the proposed bioengineering model was supported by the statistical significance of regression parameters and by the concordance between predicted and observed Periotest distributions across clinical groups.

## 3. Results

### 3.1. Descriptive Characteristics of the Study Sample

A total of 79 dental implants were included in the analysis. Among these, 37 implants were placed in patients diagnosed with systemic bone disorders, while 42 were in the control group without known bone metabolic impairment. Implants were distributed across both the maxilla and mandible, reflecting routine clinical placement conditions.

All implants included in the study were clinically stable at the time of evaluation, with no signs of overt mobility or acute peri-implant infection. Periotest measurements were successfully obtained for all implants, and no adverse events were associated with the assessment procedure.

### 3.2. Periotest Values in Patients with and Without Systemic Bone Disorders (Revizuit)

Periotest assessment revealed measurable differences in implant stability between the two study groups. Implants placed in patients with systemic bone disorders showed higher Periotest Values (PTVs) than those placed in control patients, indicating increased dynamic micromotion and reduced interface stiffness.

As summarized in Table 1, the mean PTV in the systemic bone disorder group was +2.1 ± 1.3, whereas implants in the control group demonstrated a mean PTV of −0.4 ± 1.1. The mean difference between groups was 2.5 PTV units (95% CI: 1.97–3.04), and this difference was statistically significant (*p* < 0.001), indicating a strong association between systemic bone impairment and altered biomechanical behavior at the implant–bone interface.

**Figure 1:**
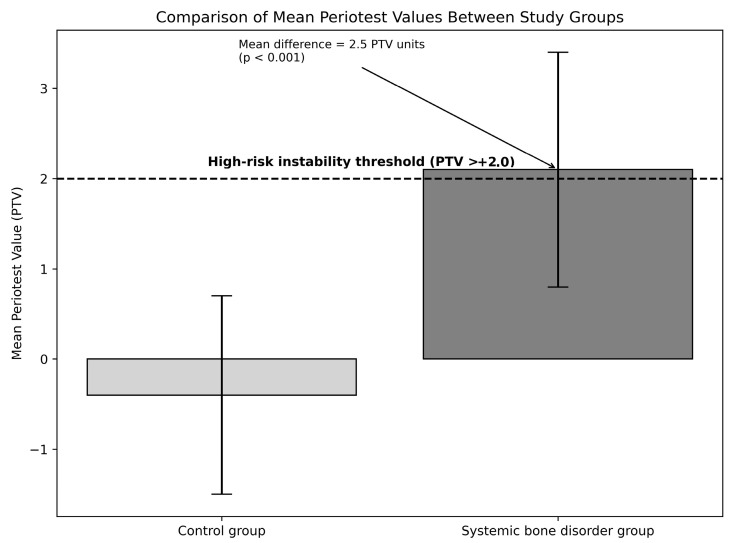
Comparison of mean Periotest values between implants placed in patients with and without systemic bone disorders.

Implants in systemically compromised patients showed significantly higher Periotest values, reflecting increased dynamic micromotion and reduced implant–bone interface stiffness, Figure 1.

### 3.3. Distribution of Implant Stability Across PTV Ranges

When Periotest values were categorized into clinically relevant stability ranges, a clear and structured distribution pattern emerged. In the control group, the majority of implants exhibited negative or low-positive Periotest values, consistent with favorable biomechanical stability and reduced implant micromotion. In contrast, implants placed in patients with systemic bone disorders were more frequently distributed in higher Periotest value ranges, reflecting increased dynamic micromotion at the implant–bone interface.

Specifically, 71% of implants in the control group had Periotest values ≤ 0, whereas only 24% of implants in the systemic bone disorder group did. Conversely, high-risk instability (PTV > +2) was observed in 46% of implants in the systemic bone disorder group, compared with only 9% in the control group.

When implant location was taken into account, maxillary implants exhibited higher Periotest values than mandibular implants in both study groups. However, implants placed in patients with systemic bone disorders consistently showed higher Periotest values across all stability categories, regardless of implant location. This finding indicates that systemic bone status was the dominant factor influencing implant micromotion, outweighing the effect of anatomical placement site (Table 2).

### 3.4. Influence of Implant Location

Implant location was associated with differences in Periotest values across the study population. In both study groups, implants placed in the maxilla had higher mean Periotest values than those placed in the mandible, indicating reduced biomechanical stability at maxillary sites.

This trend was more pronounced among patients with systemic bone disorders, in whom maxillary implants demonstrated the highest Periotest values overall. Nevertheless, when systemic bone status was included in the analysis, the presence of a systemic bone disorder remained the strongest predictor of increased Periotest values. Regardless of implant location, implants placed in systemically compromised patients consistently showed higher Periotest values than those placed in control patients, indicating that systemic bone status outweighed anatomical placement site as a determinant of implant micromotion (Table 3).

### 3.5. Predictive Bioengineering Model of Implant Instability

#### 3.5.1. Biomechanical Interface Model

Periotest values were modeled as a function of systemic bone status and implant location using a linear bioengineering framework:

(1) $$PTV=\beta_{0}+\beta_{1}\cdot B+\beta_{2}\cdot M+\beta_{3}\left(B\cdot M\right)+\varepsilon$$

where B = 1 for systemic bone disorder (0 otherwise) and M = 1 for maxillary placement (0 for mandible). Based on group mean values, the model predicted increased PTVs primarily associated with systemic bone impairment, with a secondary contribution from implant location.

In the equation, β_0_ represents the baseline Periotest value corresponding to implants placed in mandibular bone under healthy systemic conditions, β_1_ quantifies the effect of systemic bone disorders (B), β_2_ represents the effect of implant location (M), β_3_ captures the interaction between systemic bone status and implant location (B·M), and ε accounts for residual biomechanical and biological variability not explained by the model.

#### 3.5.2. Predictive Probability of High-Risk Instability

High-risk biomechanical instability was defined as PTV > +2.0. The probability of high-risk instability was estimated using a logistic predictive model:

$$P(\mathrm{Instability})=\frac{1}{1+e\left[-(\alpha_{0}+\alpha_{1}\cdot B+\alpha_{2}\cdot M)\right]}$$

where α_0_ is the intercept, α_1_ represents the systemic bone disorder effect (B), and α_2_ reflects the influence of implant location (M). Odds ratios were computed as $OR=e^{\alpha }$, and results were reported with corresponding 95% confidence intervals and *p*-values.

Systemic bone disorders were independently associated with an increased likelihood of high-risk biomechanical instability after adjustment for implant location. Maxillary placement demonstrated a more modest effect, substantially smaller than that observed for systemic bone status.

## 4. Discussion

The present study proposes a predictive bioengineering perspective on dental implant instability by integrating Periotest-derived dynamic measurements with systemic bone status and implant location. While conventional implant success criteria primarily emphasize survival and radiographic outcomes, the findings of this study support the concept that biomechanical deterioration at the implant–bone interface may precede clinically detectable failure, particularly in systemically compromised patients.

Previous experimental and clinical investigations have demonstrated that bone density and microarchitecture play a crucial role in determining implant stability and load transfer [13,14,15]. Osseodensification techniques and drilling protocols have been shown to improve primary stability in low-density bone; however, these approaches primarily address local mechanical conditions at the time of placement and may not fully compensate for long-term systemic alterations in bone metabolism [13,14]. Several clinical studies have reported that maxillary implants tend to exhibit reduced stability compared with mandibular implants due to differences in bone quality and structural stiffness [16,17]. Our findings are consistent with these observations, as maxillary placement was associated with higher Periotest values. However, the predictive model showed that systemic bone disorder status had a stronger influence on implant micromotion than anatomical location alone, suggesting that systemic bone impairment acts as a global modifier of peri-implant biomechanics across anatomical sites.

Systemic conditions such as osteoporosis are characterized not only by reduced bone mass but also by altered bone material properties and impaired mechanostat regulation [4,5,6,18]. These changes may compromise the peri-implant bone’s adaptive response to functional loading, potentially contributing to increased energy dissipation and micromotion at the implant–bone interface. Finite element and stress distribution studies have shown that reduced bone stiffness alters load transmission and increases interfacial strain concentrations [19,20], supporting the biomechanical interpretation proposed in the present model.

From a mechanical perspective, the effective stiffness of the implant–bone complex can be expressed as $k_{eff}=F/\delta$, indicating that micromotion amplitude is inversely proportional to interface stiffness. This relationship indicates that micromotion amplitude is inversely proportional to effective stiffness (δ = F/k_eff). In the context of dental implant biomechanics, reductions in bone stiffness alter load transfer and increase interfacial deformation under functional forces [19]. Experimental and theoretical studies have demonstrated that excessive micromotion at the implant–bone interface may disrupt osseointegration and compromise long-term stability [10]. Within the proposed modeling framework, systemic bone disorders are conceptualized as global modifiers of effective peri-implant stiffness. By influencing bone material properties and adaptive remodeling capacity, these conditions shift the load–micromotion relationship toward greater displacement amplitudes under comparable loading conditions, thereby increasing the probability of biomechanical instability. Furthermore, according to mechanostat theory, impaired adaptive regulation in systemically compromised bone may shift the threshold at which mechanical stimuli induce remodeling, allowing higher micromotion levels to persist [9,21,22,23,24]. Therefore, in patients with systemic bone disorders, reduced structural and material properties decrease global interface stiffness, so comparable functional loads generate greater displacement amplitudes, reflected by higher Periotest values.

Importantly, all implants included in this study were clinically stable at the time of evaluation, with no detectable macroscopic mobility. However, a substantial proportion of implants placed in patients with systemic bone disorders exhibited Periotest values within moderate- and high-risk instability ranges [24,25,26,27]. This observation supports the concept of a subclinical biomechanical instability state, in which increased micromotion exists despite the absence of overt clinical signs. Similar findings have been reported in studies comparing Periotest and resonance frequency analysis, where dynamic measurements were shown to detect subtle changes in interface behavior not captured by static assessments [16,28,29].

While resonance frequency analysis (RFA) is widely used to assess implant stability, it primarily reflects axial stiffness and boundary conditions and is most informative during early healing phases [7,8,23]. In contrast, Periotest assessment captures dynamic damping behavior and impact response, providing complementary information related to micromotion and energy dissipation [11,12,29]. The present study extends previous work by integrating Periotest values into a predictive modeling framework, rather than using them solely as descriptive indicators.

The logistic predictive model demonstrated that systemic bone disorders were associated with a 2.6-fold increase in the odds of high-risk biomechanical instability after adjustment for implant location. This finding is consistent with systematic reviews and meta-analyses reporting increased implant-related complications in patients with osteoporosis and compromised bone quality [18,21,22]. Importantly, the present approach quantifies this risk in biomechanical terms, linking systemic pathology to dynamic interface behavior rather than binary survival outcomes.

From a clinical perspective, the proposed predictive framework may support earlier identification of implants at risk for progressive instability, enabling closer monitoring, load management strategies, or preventive interventions before radiographic bone loss or clinical failure occurs. This may be particularly relevant in patients with systemic bone disorders, where subclinical micromotion can occur despite the absence of overt implant mobility. Recent clinical cohort studies have emphasized the importance of individualized risk assessment based on implant design, bone quality, and patient-specific factors [25,26,27,30]. The present findings are consistent with previous studies reporting reduced implant stability and increased biomechanical risk in patients with osteoporosis or compromised bone quality [18,21,22]. Similarly, the observed higher Periotest values in maxillary implants are consistent with the literature describing reduced bone density and structural stiffness in the maxilla compared to the mandible [16,17]. However, unlike many prior investigations that primarily evaluated implant survival rates, insertion torque, or resonance frequency analysis, the present study provides a dynamic micromotion-based interpretation through Periotest-derived predictive modeling. This bioengineering perspective extends the existing literature by linking systemic bone impairment to quantitative risk stratification rather than binary clinical outcomes. Moreover, the present findings suggest that bioengineering-informed modeling may facilitate earlier identification of implant instability in systemically compromised patients. By translating dynamic measurements into biomechanical insights, this framework may enhance risk assessment before overt clinical failure. Future prospective studies are warranted to further validate and refine this predictive approach. Several limitations should be acknowledged. The retrospective design and limited sample size may restrict generalizability, and the absence of longitudinal follow-up precludes direct assessment of long-term implant outcomes and temporal changes in biomechanical behavior. In addition, site-specific bone density measurements at the implant level were not available in the retrospective dataset. Although systemic bone disorder status was documented, quantitative local bone mineral density or volumetric bone quality parameters were not recorded, which may be important modifiers of interface stiffness and micromotion. Prosthetic loading conditions were not standardized or biomechanically quantified. Variations in occlusal scheme, restoration type, loading duration, and parafunctional habits may have influenced Periotest values and contributed to inter-individual variability. Implant stability was assessed using a single dynamic metric (Periotest value) as a proxy for complex biomechanical processes occurring at the implant–bone interface. While Periotest provides valuable information regarding micromotion and damping behavior, it does not fully capture three-dimensional stress distribution, patient-specific material properties, or finite element–derived strain patterns.

Furthermore, the proposed bioengineering framework represents a simplified conceptual model and does not incorporate patient-specific mechanical simulations, finite element stress analysis, or individualized bone material property measurements. Therefore, it should be interpreted as a predictive clinical modeling approach rather than a fully personalized biomechanical simulation.

## 5. Conclusions

This study demonstrates that dental implant instability can be interpreted as a progressive biomechanical phenomenon influenced predominantly by systemic bone disorders rather than implant location alone. Periotest-derived dynamic measurements revealed increased subclinical micromotion in patients with systemic compromise, even in the absence of clinical mobility.

By integrating Periotest values into a predictive bioengineering framework, the proposed model enables quantitative risk stratification of implant instability and highlights the dominant role of systemic bone status in determining biomechanical behavior at the implant–bone interface. This approach may support earlier identification of implants at risk for progressive instability and contribute to more individualized monitoring strategies in patients with compromised bone health.

Future prospective studies incorporating longitudinal assessment and additional biomechanical parameters are warranted to further validate and refine this predictive framework.

## Figures and Tables

**Table 1:** Comparison of Periotest values between study groups.

Study Group	Number of Implants (*n*)	Mean PTV ± SD
Control (no bone disorder)	42	−0.4 ± 1.1
Systemic bone disorder	37	+2.1 ± 1.3

Between-group comparison: Mean difference = 2.5 PTV units (95% CI: 1.97–3.04); *p* < 0.001.

**Table 2:** Distribution of dental implants across Periotest-based stability ranges according to systemic bone status.

Periotest Value (PTV) Range	Biomechanical Interpretation	Control Group, *n* (%)	Systemic Bone Disorder Group, *n* (%)
PTV ≤ 0	Low-risk stability	30 (71%)	9 (24%)
PTV 0.1–2.0	Moderate-risk instability	8 (19%)	11 (30%)
PTV > 2.0	High-risk instability	4 (9%)	17 (46%)
Total implants	—	42 (100%)	37 (100%)

**Table 3:** Influence of implant location on Periotest values according to systemic bone status.

Implant Location	Control Group (Mean PTV ± SD)	Systemic Bone Disorder Group (Mean PTV ± SD)	*p*-Value
Maxilla	+0.2 ± 1.0	+2.6 ± 1.2	<0.001
Mandible	−0.8 ± 0.9	+1.5 ± 1.1	<0.01

## Data Availability

The original contributions presented in this study are included in the article. Further inquiries can be directed to the corresponding authors.
